# Association between single-nucleotide polymorphisms and adverse events in nivolumab-treated non-small cell lung cancer patients

**DOI:** 10.1038/s41416-018-0074-1

**Published:** 2018-04-26

**Authors:** Sander Bins, Edwin A. Basak, Samira el Bouazzaoui, Stijn L. W. Koolen, E. Oomen – de Hoop, Cor H. van der Leest, Astrid A. M. van der Veldt, Stefan Sleijfer, Reno Debets, Ron H. N. van Schaik, Joachim G. J. V. Aerts, Ron H. J. Mathijssen

**Affiliations:** 1 Department of Medical Oncology, Erasmus MC Cancer Institute, Groene Hilledijk 301, Rotterdam, 3008 AE The Netherlands; 2 Department of Clinical Chemistry, Erasmus University Medical Center, ‘s-Gravendijkwal 230, Rotterdam, 3015 CE The Netherlands; 3 Department of Pulmonology, Amphia Hospital, Molengracht 21, Breda, 4818 CK The Netherlands; 4 Department of Pulmonology, Erasmus University Medical Center, ‘s-Gravendijkwal 230, Rotterdam, 3015 CE The Netherlands

**Keywords:** Non-small-cell lung cancer, Cancer immunotherapy

## Abstract

**Background:**

Treatment with PD-1 inhibitors can be hampered by severe auto-immune-related toxicities. Our objective was to identify single-nucleotide polymorphisms (SNPs) in genes previously associated with auto-immunity, which are associated with toxicities in nivolumab-treated NSCLC patients. This was in order to identify patients prone to develop severe toxicities and to gain more insight into the underlying pathobiology.

**Methods:**

We analysed 322 nivolumab-treated patients and assessed the association with toxicities for seven SNPs in four genes, which are considered contributors to PD-1-directed T-cell responses, i.e., *PDCD1*, *PTPN11*, *ZAP70* and *IFNG*. Every SNP was tested for its association with toxicity endpoints. Significant associations were tested in a validation cohort.

**Results:**

A multivariable analysis in the exploration cohort showed that homozygous variant patients for *PDCD1* 804C>T (rs2227981) had decreased odds for any grade treatment-related toxicities (*n* = 96; OR 0.4; 95% CI 0.2–1.0; *p* = 0.039). However, this result could not be validated (*n* = 85; OR 0.9; 95% CI 0.4–1.9; *p* = NS).

**Conclusions:**

Our results show that it is unlikely that the investigated SNPs have a clinical implication in predicting toxicity. A finding, even though negative, that is considered timely and instructive towards further research in biomarker development for checkpoint inhibitor treatments.

## Introduction

Tumours are able to escape from an effective immune response by exploiting phenotypical and functional changes in tumour cells as well as stromal cells that compromise infiltration, migration and local activation of anti-tumour T lymphocytes.^1^ One of these changes, representing a dominant escape mechanisms in some tumours, is over-expression of the immune checkpoint programmed cell death protein 1 (PD-1) by tumour-infiltrating CD8-positve T cells, and can be counteracted by nivolumab, thereby rescuing exhausted T cells from a non-functional status.^2,3^ Nivolumab is an anti-PD-1 antibody that is, among others, approved for the treatment of melanoma, non-small cell lung cancer (NSCLC), renal cell carcinoma, microsatellite instability-high colorectal carcinoma, and squamous cell carcinoma of the head and neck.^4–9^ Approval for other indications is expected to follow in the near future. Unfortunately, the application of anti-PD-1 blocking agents can be accompanied by severe toxicities, such as dermatitis, hypothyroidism, colitis and pneumonitis. These auto-immune-related adverse events can have extensive consequences, such as treatment discontinuation. Markers to identify patients who are at risk to develop such toxicities are key to develop a more personalised treatment approach.

**Fig. 1:**
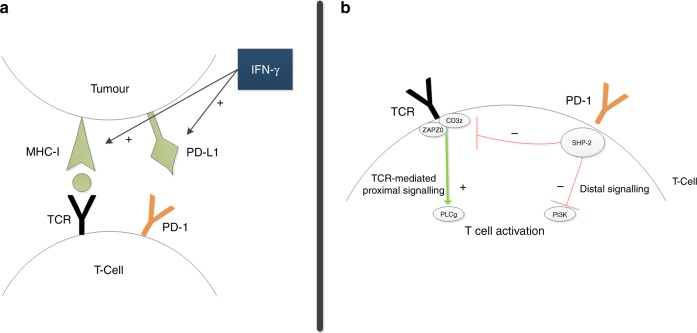
**a** Interaction between a tumour cell and a T cell. Tumour cells can activate T cells by presenting an antigen through major histocompatibility complex (MHC) to the T cell receptor (TCR). Under influence of interferon-gamma (IFNγ) tumours can express Programmed-Death ligand-1 (PD-L1), which inhibits TCR signalling by binding and activating Programmed-Death-1 (PD-1) expressed by T cells. **b** Proximal PD-1 pathway signalling. Activated PD-1 recruits SHP2, which inhibits ZAP70 function. ZAP70 is an important protein in the signalling pathway of the TCR. Complementary to its effect on ZAP70, SHP2 may also inhibit PI3K upon PD-1 activation. Both effects lead to inhibition of T cell activation. Note: the PD-1 pathway comprises many more proteins and signal transduction pathways, but these are omitted from this figure since they are not included in our analysis

Germline genetic aberrations are frequently associated with systemic toxicities as a consequence of anti-tumour agents.^10^ Hence, we argue that variants of genes involved in the PD-1 pathway are associated with the occurrence of PD-1-inhibitor-induced auto-immune-related toxicities. Moreover, PD-1, its downstream proteins SHP2 and ZAP70, and IFNγ (Fig. 1)^11–13^ are already known to be involved in disorders of the immune system: single-nucleotide polymorphisms (SNPs) in their encoding genes (*PDCD1*, *PTPN11, ZAP70* and *IFNG*) have been associated with auto-immune syndromes^14–18^ and with excessive inflammation.^19^ For example, SNPs in *PDCD1* are associated with ankylosing spondylitis and diabetes mellitus type 1, SNPs in *ZAP70* are associated with Crohn’s disease and SNPs in *PTPN11* are associated with a more severe course of inflammation after gastric infection and ulcerative colitis. Not every patient with a predisposing genotype develops an auto-immune phenotype, but the sensitivity of the PD-1 axis, or its activity, might be altered in those persons. When treated with PD-(L)1 inhibiting drugs, these (asymptomatic) carriers of an aberrant genotype might be more prone to develop immune related adverse events than patients with a wildtype PD-1 axis. Hence, we hypothesised that additional PD-1 inhibition by nivolumab would trigger auto-immunity and consequently lead to more toxicity in patients who harbor germline genetic polymorphisms in the PD-1 axis. In this study, we explored a large cohort of nivolumab-treated NSCLC patients in daily clinical practice and studied whether patients with SNPs in the PD-1 and PD-1-related genes experienced toxicity more (or less) frequently or severely than other patients.

## Materials and methods

### Study design

We collected data from NSCLC patients who started nivolumab monotherapy treatment, without other concomitant anti-cancer agents (e.g., chemotherapy), at two large Dutch hospitals (the Erasmus MC Cancer Institute, Rotterdam, and at the Amphia Hospital, Breda) between July 26th 2013 and April 5th 2017. Clinical data was collected until June 1st 2017. Patients from whom whole blood for DNA analysis was (prospectively) collected were included in this study (local ethics board study number MEC 02-1002). Patients were ranked based on date of treatment start, thereafter patients were alternately allocated to an exploration cohort or a validation cohort, each consisting of 161 patients.

Patient characteristics were collected from the hospitals electronic patient record systems and included demographic and clinical information (e.g., age at start of treatment, gender, ethnicity, WHO performance status at start of treatment, previous anti-tumour treatments, treatment interruptions, NSCLC subtype and toxicities). WHO performance status was determined by judgment of the clinician at the nearest time point before start of nivolumab treatment, and was regarded as “missing” if this point was more than 1 month before treatment start. Concomitant use of oral or intravenous corticosteroids to resolve immune-related toxicities was also recorded and regarded as a surrogate for adverse events.

Adverse events from start of treatment until end of follow-up were retracted from the patient status, and best corresponding grade was retrieved according to National Cancer Institute Common Terminology Criteria for Adverse Events (NCI-CTCAE) v4.03 if not already graded appropriately in the patient status. Adverse events that were possibly, probably, or definitely related to the nivolumab treatment were classified as treatment-related adverse events. The diagnosis of hepatitis was based on judgment of the treating clinician. An adverse event was considered pre-existent if it was present in the same or higher degree before treatment start, and therefore not considered as an event.

### Selection of SNPs

We selected seven SNPs in the *PDCD1*, *PTPN11*, *ZAP70* and *IFNG* genes for analysis (see Table 1 for details). SNPs with a reported minor allele frequency (MAF) above 5% were included.

**Table 1:** Investigated single-nucleotide polymorphisms

Gene	Protein	rs-number	Variant	Assay ID	Cohort	WT	HET	HVAR	Undetermined	MAF	HWE^a^
*PDCD1*	PD-1	rs2227981	804C>T	c__57931286_20	Exploration	50	76	34	1	45%	0.61
					Validation	44	78	39	0	48%	0.70
		rs2227982	644C>T	c__57931287_10	Exploration	159	2	0	0	1%	0.9
					Validation	161	0	0	0	0%	n/a
		rs10204525	*889G>A	c____172862_10	Exploration	139	20	2	0	7%	0.21
					Validation	132	27	2	0	10%	0.65
*PTPN11*	SHP2	rs2301756	333–223A>G	c___2978562_20	Exploration	131	30	0	0	9%	0.19
					Validation	128	30	3	0	11%	0.43
*ZAP70*	ZAP70	rs13420683	−21–4127C>A	c___1278468_10	Exploration	89	55	13	4	26%	0.29
					Validation	88	51	20	2	29%	0.007
*IFNG*	IFNγ	rs2069718	367–895C>T	c__15799728_10	Exploration	57	74	30	0	42%	0.49
					Validation	55	74	30	2	42%	0.57
		rs2069705	−1616T>C	c__15944115_20	Exploration	72	70	19	0	34%	0.75
					Validation	73	65	23	0	34%	0.18

* WT* wildtype, *HET* heterozygous, *HVAR* homozygous variant, *MAF* minor allele frequency, *HWE* Hardy–Weinberg equilibrium.

^a^ If <0.05 not consistent with HWE

### DNA isolation

Four hundred microliters of whole-blood specimens were collected in EDTA tubes and DNA was extracted in a final elution volume of 200 μL using the MagNAPure Compact instrument (Roche Diagnostics GmbH, Mannheim, Germany) and the Nucleic Acid Isolation Kit I (Roche Diagnostics GmbH).

### Taqman genotyping

Genotyping was performed using predesigned DME Taqman allelic discrimination assays on the Life Technologies Taqman 7500 system (Applied Biosystems, Life Technologies Europe BV, Bleiswijk, The Netherlands; Table 1). Each assay consisted of two allele-specific minor groove binding (MGB) probes, labeled with the fluorescent dyes VIC and FAM. Polymerase chain reactions (PCR) were performed in a reaction volume of 10 µL, containing assay-specific primers, allele-specific Taqman MGB probes (Applied Biosystems), Abgene Absolute QPCR ROx Mix (Thermo Scientific, Life Technologies Europe BV, Bleiswijk, The Netherlands) and genomic DNA (20 ng). The thermal profile consisted of 40 cycles of denaturation at 95 °C for 20 s, annealing at 92 °C for 3 s and extension at 60 °C for 30 s. Genotypes were scored by measuring allele-specific fluorescence using the 7500 software v2.3 for allelic discrimination (Applied Biosystems).

### Statistics

Distribution of genotypes was tested for the Hardy–Weinberg equilibrium (HWE) using the *χ*^2^ test (Table 1). Since *ZAP70*-32-4127C>A was not in HWE in the validation cohort, this SNP was excluded from further analyses. SNPs with a MAF <5% in our cohort were also excluded from further analyses, which was the case for *PDCD1* 644C>T. Linkage disequilibrium (LD) analyses were performed using SNAP.^20^ Because none of the analysed SNP pairs met our preset criteria for LD (R2 > 0.8), all SNPs were analysed individually. For every SNP, the best fitting model (that is, the model resulting in the best association) was selected from four models, i.e., a dominant, recessive, additive, and multiplicative model.^21^ The dominant and recessive models were used to test associations between SNPs and toxicity, steroid use, and temporary or definitive treatment discontinuation due to toxicity using the *χ*^2^ test or, in case one of the observed numbers was ≤10 or one of the expected numbers was <5, with Fisher’s exact test. The additive and multiplicative models were used to test the SNPs in logistic regression as ordinal and linear predictor variables, respectively. If a SNP was associated with toxicity with *p* < 0.1, it was entered in a multivariable logistic regression model together with age and gender in order to avoid bias by those parameters. Multivariable analysis was only performed in case of approximately 10 or more events per assessed variable in order to avoid bias of the regression coefficients.^22^ Models with a significant outcome in univariable or multivariable analysis in the exploration cohort were examined in the validation cohort. SPSS software v21 (SPSS, Chicago, IL, USA) was used for the above-mentioned analyses. A two-sided *p* < 0.05 was regarded as significant and no correction for multiple testing was applied.

## Results

### Patient characteristics

Blood samples for DNA analysis were available for 322 NSCLC patients in total, of whom 63% (*n* = 202) were male, having a mean age at start of therapy of 65 years, and 58% (*n* = 188) had a WHO performance status of 1 at start. More baseline characteristics for both the exploration and validation cohorts are depicted in Table 2. All characteristics were comparable between both groups (*t*-test). All patients were treated with the standard dose of nivolumab at 3 mg/kg Q2W that was not changed during treatment. Median absolute starting dose was 222 mg (IQR 192–263 mg) and median duration of follow-up was 126 days (IQR 70–214 days).

**Table 2:** Patient characteristics at baseline

	Number of patients (*n* = 161; exploration cohort)	Number of patients (*n* = 161; validation cohort)
Gender		
Male	108 (67%)	94 (58%)
Female	53 (33%)	67 (42%)
Age at start (years)		
Mean (±SD)	64 (±8.1)	65 (±9.1)
WHO performance status		
0	26 (16%)	28 (17%)
1	95 (59%)	93 (58%)
2	2 (1%)	5 (3%)
3	1 (1%)	0 (0%)
Unknown	37 (23%)	35 (22%)
Primary lung tumour		
Adenocarcinoma	97 (60%)	101 (63%)
Squamous cell carcinoma	47 (29%)	49 (30%)
Great cell carcinoma	15 (9%)	7 (4%)
Unspecified	2 (1%)	4 (3%)
Number of pre-treatment lines		
0	1 (1%)	0 (0%)
1	104 (65%)	113 (70%)
2	44 (27%)	38 (24%)
>2	12 (7%)	10 (6%)
Ethnicity		
Caucasian	152 (94%)	156 (97%)
Other	4 (3%)	1 (1%)
Unknown	5 (3%)	4 (3%)

### Adverse events

Fifty patients (16%) had any grade ≥3 toxicity (Table 3 and Supplementary Table S1). When analysing all-grade toxicity that was not pre-existent, hypothyroidism or hyperthyroidism (*n* = 129; 40%), elevated transaminases (*n* = 106; 33%), and skin toxicity (*n* = 46; 14%) occurred most frequently. Treatment-related grade ≥3 toxicities occurred in 23 patients (7%). Any grade treatment-related adverse events occurred in 181 patients (56%; Supplementary Table S2).

**Table 3:** Frequencies of toxicity endpoints corrected for pre-existence

	Number of patients (*n* = 161; exploration cohort)	Number of patients (*n* = 161; validation cohort)
Any grade toxicity		
Diarrhea	6 (4%)	7 (4%)
Skin toxicity	23 (14%)	23 (14%)
Elevated transaminases	50 (31%)	56 (35%)
Elevated bilirubin	7 (4%)	7 (4%)
Hepatitis	6 (4%)	2 (1%)
Hypothyroidism or hyperthyroidism	64 (40%)	65 (40%)
Pneumonitis/Interstitial lung disease	8 (5%)	8 (5%)
Colitis	2 (1%)	4 (3%)
Rheumatological toxicity	10 (6%)	13 (8%)
Hypophysitis	1 (1%)	1 (1%)
Treatment-related toxicity	96 (60%)	85 (53%)
Other outcomes		
Decreased renal clearance grade ≥2	28 (17%)	41 (26%)
Any grade 3 or higher toxicity	24 (15%)	26 (16%)
Any grade 3 or higher treatment-related toxicity	14 (9%)	9 (6%)
Steroid use	42 (26%)	38 (24%)
Treatment stop caused by toxicity		
Temporary interruption	15 (9%)	15 (9%)
Definitive discontinuation	6 (4%)	5 (3%)

Eighty patients (25%) required oral or intravenous steroids during nivolumab treatment. Thirty patients (9%) temporarily interrupted nivolumab treatment because of toxicities, whereas eleven patients (3%) had to permanently stop treatment because of toxicities.

### Treatment details

At the end of the data collection period, 150 patients (47%) were still being treated with nivolumab. A total of 42 patients (13%) stopped because of clinical deterioration, 108 patients (34%) stopped after progressive disease (assessed by the treating clinician), eleven patients (3%) stopped because of treatment-related toxicities and ten patients (3%) stopped because of other reasons, i.e., due to patients’ request (*n* = 5), sudden death (*n* = 4) or a non-measurable lesion after re-evaluation of the baseline CT-scan (*n* = 1). Twenty-three patients (7%) were lost to follow-up.

### Association of SNPs with toxicity during nivolumab treatment

All outcomes of the exploration cohort that occurred in a sufficient number of patients for univariable analysis are shown in Supplementary Table S3. Those outcomes with an association of *p* < 0.1 in relation to the concerning SNP are described in Table 4. The *PTPN11* 333–223A>G and *PDCD1* *889G>A SNPs were only tested in a dominant model since the number of homozygous mutant patients was too low for reliable analysis. Since skin toxicity, rheumatological toxicity, any grade ≥3 toxicity, treatment related grade ≥3 toxicity, and temporary stop caused by toxicity occurred in an insufficient number of patients for reliable multivariable analysis, these outcomes were only analysed univariably. Diarrhea, elevated bilirubin, hepatitis, pneumonitis, neuropathy, colitis and permanent stop caused by toxicity were ineligible for univariable analysis. Decreased renal clearance (≥grade 2), elevated transaminases, hypothyroidism or hyperthyroidism and treatment-related adverse events (any grade) were studied in multivariable analyses.

**Table 4:** Association between SNPs with *p* < 0.1 in the univariable analysis and endpoints (exploration cohort)

Endpoint	Factor	Genotype	Univariable		Multivariable	
			OR (95% CI)	*p*-value	OR (95% CI)	*p*-value
Decreased renal clearance (≥grade 2)	Age				1.056 (0.999–1.116)	0.056
	Gender (f. vs. m.)				1.364 (0.555–3.350)	0.498
	*PDCD1* 804C>T	CC → CT → TT ^a^	1.631 (0.917–2.903)	0.096^b^	1.545 (0.864–2.763)	0.142
Elevated transaminases (any grade)	Age				1.010 (0.967–1.055)	0.649
	Gender (f. vs. m.)				1.741 (0.841–3.604)	0.135
	*PTPN11* 333–223A>G	GG + AG vs. AA	2.309 (1.024–5.208)	0.041^c^	2.421 (1.061–5.523)	0.036
Hypothyroidism or hyperthyroidism (any grade)	Age				0.989 (0.950–1.030)	0.598
	Gender (f. vs. m.)				1.158 (0.579–2.315)	0.678
	*PTPN11* 333–223A>G	GG + AG vs. AA	0.395 (0.158–0.985)	0.061^d^	0.403 (0.161–1.007)	0.052
Treatment-related adverse events (any grade)	Age				1.007 (0.967–1.049)	0.738
	Gender (f. vs. m.)				1.234 (0.609–2.498)	0.560
	*PDCD1* 804C>T	TT vs. CC + CT	0.454 (0.211–0.978)	0.041^c^	0.440 (0.202–0.958)	0.039
Rheumatological toxicity (any grade)	*IFNG* -1616T>C^e^	CC vs. TT + TC	6.044 (1.531–23.857)	0.019^d^		

*OR* odds ratio, *CI* confidence interval, *f. vs. m.* females versus males.

^a^Additive model was used.

^b^Logistic regression was used.

^c^Chi-square test was used.

^d^Fisher’s Exact test was used.

^e^Only tested univariably due to the number of events

In the exploration cohort, homozygous variant patients for *PDCD1* 804C>T had decreased odds for developing any grade treatment-related adverse events (OR 0.4; 95% CI 0.2–1.0; *p* = 0.039). At least one variant allele at *PTPN11* 333–223A>G was associated with increased odds for elevated transaminases (OR 2.4; 95% CI 1.1–5.5; *p* = 0.036). The relationship between hypothyroidism or hyperthyroidism and *PTPN11* 333–223A>G in univariable analysis did not hold in multivariable analysis (OR 0.4; 95% CI 0.2–1.0; *p* = 0.052). Of the endpoints that could only be analysed univariably due to the number of patients, *IFNG* −1616T>C was associated with rheumatological toxicity (OR 6.0; 95% CI 1.5–23.9; *p* = 0.019).

Associations between SNPs and endpoints that showed significance when tested in the exploration cohort, were re-tested in the validation cohort of which results are shown in Table 5. Since the associations between *PTPN11* 333–223A>G and elevated transaminases (OR 1.509; 95% CI 0.690–3.300; *p* = 0.301), and between *PDCD1* 804C>T and any grade treatment-related adverse events (OR 0.923; 95% CI 0.449–1.899; *p* = 0.828) had *p*-values > 0.1, these were not considered for multivariable analysis in the validation cohort. Furthermore, the above described association between *IFNG* -1616T>C and rheumatological toxicity in univariable analysis did not hold in the validation cohort (OR 0.5; 95% CI 0.1–3.9; *p* = 0.695).

**Table 5:** Association between SNPs and endpoints with a significant correlation in the exploration cohort

Endpoint	Factor	Genotype	Univariable	
			OR (95% CI)	*p*-value
Elevated transaminases (any grade)	*PTPN11* 333–223A>G	GG + AG vs. AA	1.509 (0.690–3.300)	0.301^a^
Treatment-related adverse events (any grade)	*PDCD1* 804C>T	TT vs. CC + CT	0.923 (0.449–1.899)	0.828^a^
Rheumatological toxicity (any grade)	*IFNG* -1616T>C	CC vs. TT + TC	0.477 (0.059–3.857)	0.695^b^

*OR* odds ratio, *CI* confidence interval.

^a^*χ*^2^ test was used.

^b^Fisher’s Exact test was used

## Discussion

Although this is—to the best of our knowledge—the largest pharmacogenetic pathway analysis in nivolumab treated NSCLC patients so far, we could not validate SNPs of the PD-1 or PD-1-related genes relevant for toxicity. Nonetheless, in the exploration cohort, the TT genotype in the *PDCD1* 804C>T SNP (rs2227981) was associated with less nivolumab related toxicity. Moreover, at least one G allele in the *PTPN11* 333–223A>G SNP (rs2301756) was associated with an increased risk for developing elevated transaminases. Also, in univariable analysis, homozygous mutant patients for the *IFNG* -1616T>C SNP had higher odds for developing rheumatological toxicity. However, all findings could not be replicated in the validation cohort.

Interestingly, a variant allele of *PDCD1* 804C>T has previously been associated with susceptibility for ankylosing spondylitis and type I diabetes.^14^ Possibly, this could be related to a decreased activity profile of the PD-1 pathway in patients harboring this SNP, and these patients may therefore experience less inhibition of T cell activation (Fig. 1). It may seem paradoxical that harboring two variant alleles in the rs2227981 SNP is associated with less toxicity in the exploration cohort, whereas earlier findings relate it to a higher incidence of auto-immune related events. Speculating on this, it could be that patients with the variant allele already have less PD-1-mediated T cell inhibition, which may make this pathway less susceptible to blockade by nivolumab. Consequently, these patients may experience fewer treatment-related toxicities than wildtype patients.

Our finding in the exploration cohort that patients with at least one variant allele in the rs2301756 SNP have an increased risk for developing elevated transaminases is in line with earlier studies: the variant allele has previously been associated with susceptibility for ulcerative colitis in the Japanese population^18^ and with an increased risk for gastric atrophy after *H. Pylori* infection,^19^ possibly caused by chronic inflammation. Our finding supports the notion that genetic variants in the SHP2 gene (*PTPN11*) prevent T cell suppression and do result in stronger T cell reactions, in particular when there is additional inhibition of PD-1 via nivolumab treatment. Speculating on the underlying biological mechanism, we argue that reduced SHP2 activity—in case of the above-mentioned SNP—results in less de-phosphorylation (and thereby de-activation) of its targets ZAP-70 and PI3K. As such, the ‘brake’ on proximal TCR-mediated signalling as well as more distal PI3K signalling is relieved, which then results in enhanced T cell activation (see Fig. 1).

In addition, the earlier reported finding between SNPs in *IFNG* with systemic lupus erthythaematosis^17^ corresponds with our finding in the exploration cohort, in which this SNP is associated with rheumatological toxicities. The rs2069718 SNP in *IFNG*, and the rs10204525 SNP in *PDCD1*, located on two genes that affect each other’s expression according to a negative feedback loop, were not associated with outcomes in both cohorts, possibly due to the influence of other adjacent pathways related to IFNγ and PD-1. We did not explore these adjacent pathways in the current analysis, however, this research is part of ongoing work.

The explorative nature of this study and the retrospective data collection may result in a slightly different low-grade toxicity profile than reported previously.^23^ Nevertheless, the clinically most relevant endpoints, i.e., any grade ≥3 toxicity and treatment-related grade ≥3 toxicity, are unlikely to be underrepresented in this study as the median follow-up time (>4 months) exceeds the previously described expected time for toxicities to occur.^24^ In fact an endpoint such as treatment-related adverse events eliminates distortion of the association between a SNP and an endpoint by excluding adverse events that could be a consequence of other factors, such as disease progression. Furthermore, some endpoints, e.g., colitis, occurred too infrequent to be assessed in multivariable analysis, which requires about ten patients per assessed variable.^22^ The cutoff date resulted in a relatively short follow-up time in a small number of patients in both cohorts (*n* = 13 versus *n* = 12, respectively). However, earlier trials described that most relatively frequent adverse events occur during the first few weeks of treatment, such as skin, gastrointestinal, and hepatic toxicities.^25^ Therefore, we expect that inclusion of the concerning patients had a negligible effect on most important endpoints.

Associations between SNPs and survival are also important, since predictive biomarkers for survival are currently lacking. As the survival data are not mature enough yet, we will assess these associations once the data allow us to. Although germline genetics might seem logical biomarkers for general systemic effects, such as adverse events, and although (local) antitumour T cell effects are also affected by somatic mutations and mutational load,^26^ prior treatment, and possibly even the microbiome,^27^ SNPs studied here might provide additional predictive information to these other biomarkers.

Despite our above stated explanations for the findings in the exploration cohort, it should be emphasised that they could not be validated, even though the distribution of events per genotype was comparable between the two cohorts for the relationships between elevated transaminases and the SNP in *PTPN11* and between any grade treatment-related adverse events and the SNP in *PDCD1* 804. However, in the validation cohort, there was one patient with a rheumatological toxicity in the homozygous mutant group for the *IFNG* -1616T>C SNP, which could explain the contradictory finding with the exploration cohort.

The current genotyping effort should not be considered comprehensive. Even though it is likely that other factors than germline genetics may contribute to the occurrence of adverse events, our study provides a starting point from where other genes, or even other SNPs in the investigated genes, could be investigated towards future biomarker development for anti-PD-1 treatment outcomes.

As mentioned, main limitations of this analysis arise from the retrospective data collection. Therefore, subtle adverse events (grade 2 or less) may not have been mentioned by the treating physician, or its pre-existence may not have been noticed. Also, the distinction between treatment-related toxicities or adverse events caused by other sources may be unclear for some lower-grade and general toxicities. Moreover, since some toxicities occur only in a very small proportion of immunotherapy treated patients, these outcomes had to be excluded from the analysis. Finally, this analysis was restricted to a limited proportion of the PD-1 pathway and its associated genes and polymorphisms.

In conclusion, this study shows that future biomarker research for nivolumab treatment outcomes can and should embrace germline genetics. However, it is unlikely that the investigated SNPs have a clinical implication in the prediction of toxicity in NSCLC patients treated with nivolumab. Considering underlying biological mechanisms, these SNP probably have no relevance in patients with other tumour types and patients receiving other anti-PD-1 agents. The reported lack of associations facilitates further research in biomarker development for checkpoint inhibitor treatments.

## Electronic supplementary material

Table S1

Table S2

Table S3

## Availability of data and materials

Data supporting the results are archived in the Erasmus Medical Center.
