# Supplementary material for: Association between single-nucleotide polymorphisms and adverse events in nivolumab-treated non-small cell lung cancer patients
Source: Br J Cancer. 2018 Apr 26;118(10):1296–301. doi: 10.1038/s41416-018-0074-1 (PMC5959881; doi:10.1038/s41416-018-0074-1)
Supplement: Supplementary file 1 — Table S1 [file 41416_2018_74_MOESM1_ESM.docx]

| **Table S1. Any grade 3 or higher adverse events** | | |
| --- | --- | --- |
| **Any ≥grade 3 adverse events** | **Number of patients (n=24; exploration cohort)** | **Number of patients (n=26; validation cohort)** |
| Diarrhea | 1 | 1 |
| Colitis | 1 | 3 |
| Skin toxicity | 2 | 1 |
| Elevated transaminases | 5 | 3 |
| Elevated bilirubin | 0 | 1 |
| Decreased renal clearance | 3 | 8 |
| Hypothyroidism or hyperthyroidism | 0 | 1 |
| Hypophysitis | 1 | 1 |
| Pneumonitis | 3 | 3 |
| Dyspnea | 5 | 5 |
| Anemia | 1 | 0 |
| Pleural effusion | 1 | 0 |
| Leukocytosis | 1 | 0 |
| Fatigue | 2 | 3 |
| Anaphylaxis | 1 | 0 |
| Neuropathy | 0 | 1 |
| Nephritis | 0 | 2 |
| Adrenal insufficiency | 0 | 1 |
| Constipation | 0 | 1 |
| Fever | 0 | 1 |
