# Supplementary material for: Association between single-nucleotide polymorphisms and adverse events in nivolumab-treated non-small cell lung cancer patients
Source: Br J Cancer. 2018 Apr 26;118(10):1296–301. doi: 10.1038/s41416-018-0074-1 (PMC5959881; doi:10.1038/s41416-018-0074-1)
Supplement: Supplementary file 2 — Table S2 [file 41416_2018_74_MOESM2_ESM.docx]

| **Table S2. Treatment-related adverse events** | | | |
| --- | --- | --- | --- |
| **Any treatment related adverse event** | **Grade** | **Number of patients (n=96; exploration cohort)** | **Number of patients (n=85; validation cohort)** |
| Diarrhea | 1 | 4 | 2 |
|  | 2 | 1 | 4 |
|  | 3 | 1 | 1 |
| Colitis | 1 | 1 | 0 |
|  | 2 | 0 | 1 |
|  | 3 | 1 | 3 |
| Skin toxicity | 1 | 13 | 10 |
|  | 2 | 8 | 12 |
|  | 3 | 2 | 1 |
| Hepatitis | 1 | 0 | 1 |
|  | 2 | 1 | 1 |
|  | 3 | 5 | 0 |
| Hypothyroidism or hyperthyroidism | 1 | 53 | 51 |
|  | 2 | 11 | 13 |
|  | 3 | 0 | 1 |
| Hypophysitis | 3 | 1 | 1 |
| Pneumonitis | 1 | 3 | 1 |
|  | 2 | 2 | 4 |
|  | 3 | 2 | 3 |
|  | 5 | 1 | 0 |
| Rheumatological toxicity | 1 | 7 | 4 |
|  | 2 | 3 | 9 |
| Tendinitis | 1 | 0 | 1 |
| Nephritis | 3 | 0 | 2 |
| Adrenal insufficiency | 3 | 0 | 1 |
| Chondritis | 2 | 1 | 0 |
| Diverticulitis | 2 | 1 | 0 |
| Myalgia | 1 | 1 | 0 |
|  | 2 | 2 | 0 |
| Stomatitis | 1 | 1 | 0 |
| Vasculitis | 2 | 1 | 1 |
| Conjunctivitis | 2 | 1 | 0 |
| Bursitis | 1 | 1 | 0 |
| Balanitis | 1 | 1 | 0 |
| Pemphigus | 1 | 1 | 0 |
| Toxic keratopathy | 2 | 1 | 0 |
| Anemia | 3 | 1 | 0 |
| Leukocytosis | 3 | 1 | 0 |
