# Supplementary material for: Association between single-nucleotide polymorphisms and adverse events in nivolumab-treated non-small cell lung cancer patients
Source: Br J Cancer. 2018 Apr 26;118(10):1296–301. doi: 10.1038/s41416-018-0074-1 (PMC5959881; doi:10.1038/s41416-018-0074-1)
Supplement: Supplementary file 3 — Table S3 [file 41416_2018_74_MOESM3_ESM.docx]

| **Table S3. Association between all investigated SNPs and endpoints in the exploration cohort** | | | | |
| --- | --- | --- | --- | --- |
| **Endpoint** | **Factor** | **Genotype** | **Univariable** | |
|  |  |  | **OR (95% CI)** | **p-value** |
| Skin toxicity  (any grade) | *PDCD1* 804C>T | TT + CT vs. CC | 0.611 (0.242 – 1.540) | 0.293^a^ |
|  | *PDCD1 **889G>A | AA + GA vs. GG | 0.939 (0.254 – 3.470) | 1.000^b^ |
|  | *PTPN11* 333-223A>G | GG + AG vs. AA | 0.907 (0.284 – 2.892) | 1.000^b^ |
|  | *IFNG* 367-895C>T | TT + CT vs. CC | 0.830 (0.335 – 2.056) | 0.686^a^ |
|  | *IFNG* -1616T>C | CC + TC vs. TT | 0.864 (0.357 – 2.093) | 0.746^a^ |
| Elevated transaminases (any grade) | *PDCD1* 804C>T | TT + CT vs. CC | 0.698 (0.343 – 1.421) | 0.320^a^ |
|  | *PDCD1* *889G>A | AA + GA vs. GG | 0.810 (0.297 – 2.210) | 0.807^b^ |
|  | *PTPN11* 333-223A>G | GG + AG vs. AA | 2.309 (1.024 – 5.208) | 0.041^a^ |
|  | *IFNG* 367-895C>T | CC 🡪 CT 🡪 TT^c^ | 0.767 (0.477 – 1.233) | 0.274^d^ |
|  | *IFNG* -1616T>C | CC + TC vs. TT | 0.581 (0.296 – 1.138) | 0.112^a^ |
| Decreased renal clearance (≥grade 2) | *PDCD1* 804C>T | CC 🡪 CT 🡪 TT^c^ | 1.631 (0.917 – 2.903) | 0.096^d^ |
|  | *PDCD1 **889G>A | AA + GA vs. GG | 1.483 (0.497 – 4.425) | 0.544^b^ |
|  | *PTPN11* 333-223A>G | GG + AG vs. AA | 1.239 (0.453 – 3.384) | 0.789^b^ |
|  | *IFNG* 367-895C>T | CC 🡪 CT 🡪 TT^c^ | 1.153 (0.654 – 2.030) | 0.623^d^ |
|  | *IFNG* -1616T>C | TT 🡪 TC 🡪 CC^c^ | 0.695 (0.416 – 1.162) | 0.165^d^ |
| Hypothyroidism or hyperthyroidism (any grade) | *PDCD1* 804C>T | CC + CT vs. TT | 0.556 (0.245 – 1.259) | 0.156^a^ |
|  | *PDCD1* *889G>A | AA + AG vs. GG | 0.524 (0.193 – 1.419) | 0.245^b^ |
|  | *PTPN11* 333-223A>G | GG + AG vs. AA | 0.395 (0.158 – 0.985) | 0.061^b^ |
|  | *IFNG* 367-895C>T | CC 🡪 CT 🡪 TT^c^ | 1.148 (0.739 – 1.785) | 0.539^d^ |
|  | *IFNG* -1616T>C | TT 🡪 TC 🡪 CC^c^ | 1.332 (0.835 – 2.125) | 0.229^d^ |
| Rheumatological toxicity (any grade) | *PDCD1* 804C>T | CC 🡪 CT 🡪 TT^e^ | 0.475 (0.047 – 4.766) | 0.628^d^ |
|  | *PDCD1* *889G>A | AA + GA vs. GG | 0.688 (0.083 – 5.712) | 1.000^b^ |
|  | *PTPN11* 333-223A>G | GG + AG vs. AA | 0.467 (0.057 – 3.837) | 0.690^b^ |
|  | *IFNG* 367-895C>T | CC + CT vs. TT | 3.205 (0.844 – 12.165) | 0.091^b^ |
|  | *IFNG* -1616T>C | TT + TC vs. CC | 6.044 (1.531 – 23.857) | 0.019^b^ |
| Any grade ≥3 toxicity | *PDCD1* 804C>T | TT + CT vs. CC | 0.829 (0.326 – 2.105) | 0.808^b^ |
|  | *PDCD1* *889G>A | AA + GA vs. GG | 1.858 (0.613 – 5.627) | 0.330^b^ |
|  | *PTPN11* 333-223A>G | GG + AG vs. AA | 1.569 (0.564 – 4.368) | 0.399^b^ |
|  | *IFNG* 367-895C>T | TT + CT vs. CC | 0.489 (0.204 – 1.175) | 0.105^a^ |
|  | *IFNG* -1616T>C | CC + TC vs. TT | 0.640 (0.268 – 1.530) | 0.313^a^ |
| Treatment-related adverse events (any grade) | *PDCD1* 804C>T | CC + CT vs. TT | 0.454 (0.211 – 0.978) | 0.041^a^ |

| **Table S3. Association between all investigated SNPs and endpoints in the exploration cohort (continued)** | | | | |
| --- | --- | --- | --- | --- |
| **Endpoint** | **Factor** | **Genotype** | **Univariable** | |
|  |  |  | **OR (95% CI)** | **p-value** |
| Treatment-related adverse events (any grade) | *PDCD1* *889G>A | AA + GA vs. GG | 0.635 (0.258 – 1.567) | 0.322^a^ |
|  | *PTPN11* 333-223A>G | GG + AG vs. AA | 0.729 (0.325 – 1.619) | 0.436^a^ |
|  | *IFNG* 367-895C>T | CC + CT vs. TT | 1.742 (0.742 – 4.093) | 0.222^b^ |
|  | *IFNG* -1616T>C | TT + TC vs. CC | 2.049 (0.700 – 5.997) | 0.220^b^ |
| Treatment-related adverse events (≥grade 3) | *PDCD1* 804C>T | TT + CT vs. CC | 0.354 (0.113 – 1.116) | 0.114^b^ |
|  | *PDCD1* *889G>A | AA + GA vs. GG | 1.058 (0.220 – 5.084) | 1.000^b^ |
|  | *PTPN11* 333-223A>G | GG + AG vs. AA | 1.862 (0.542 – 6.398) | 0.298^b^ |
|  | *IFNG* 367-895C>T | TT + CT vs. CC | 0.515 (0.171 – 1.551) | 0.252^b^ |
|  | *IFNG* -1616T>C | TT 🡪 TC 🡪 CC^c^ | 0.643 (0.266 – 1.554) | 0.326^d^ |
| Steroid use | *PDCD1* 804C>T | TT + CT vs. CC | 0.625 (0.297 – 1.314) | 0.213^a^ |
|  | *PDCD1* *889G>A | AA + GA vs. GG | 1.073 (0.390 – 2.952) | 1.000^b^ |
|  | *PTPN11* 333-223A>G | GG + AG vs. AA | 0.835 (0.329 – 2.117) | 0.820^b^ |
|  | *IFNG* 367-895C>T | CC + CT vs. TT | 0.660 (0.249 – 1.746) | 0.493^b^ |
|  | *IFNG* -1616T>C | CC + TC vs. TT | 1.108 (0.545 – 2.252) | 0.778^a^ |
| Temporary stop caused by toxicity | *PDCD1* 804C>T | TT + CT vs. CC | 0.482 (0.164 – 1.412) | 0.240^b^ |
|  | *PDCD1* *889G>A | AA + GA vs. GG | 0.969 (0.203 – 4.620) | 1.000^a^ |
|  | *PTPN11* 333-223A>G | GG + AG vs. AA | 1.102 (0.291 – 4.176) | 1.000^a^ |
|  | *IFNG* 367-895C>T | TT + CT vs. CC | 0.805 (0.271 – 2.389) | 0.779^b^ |
|  | *IFNG* -1616T>C | CC + TC vs. TT | 0.683 (0.235 – 1.983) | 0.588^b^ |

*Abbreviations*: OR, odds ratio; CI, confidence interval.

^a^ Chi-square test was used; ^b^ Fisher’s Exact test was used; ^c^Additive model was used; ^d^Logistic regression was used; ^e^Multiplicative model was used
